# Supplementary figures and images for: The Serological Cross-Detection of Bat-Borne Hantaviruses: A Valid Strategy or Taking Chances?
Source: Viruses. 2021 Jun 22;13(7):1188. doi: 10.3390/v13071188 (PMC8309984; doi:10.3390/v13071188)

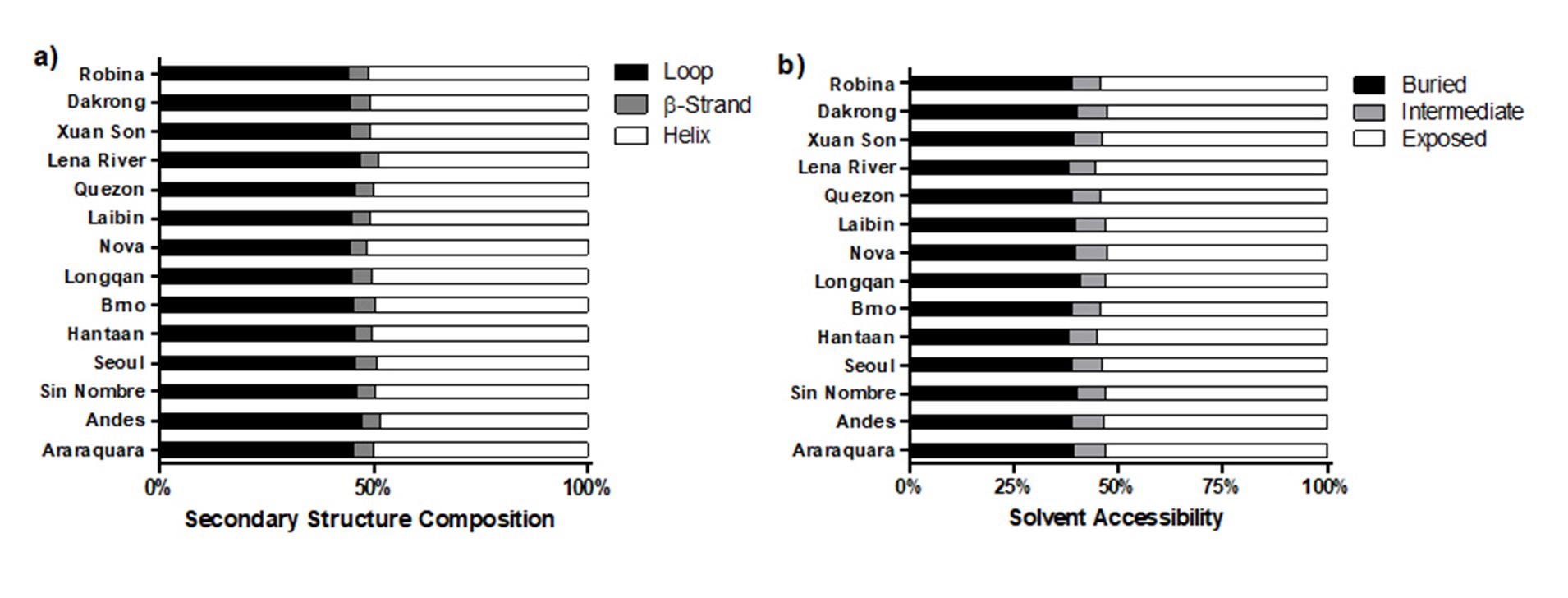

Supplement: Supplementary file 1 [file viruses-13-01188-s001.zip › Figure S2.jpg]

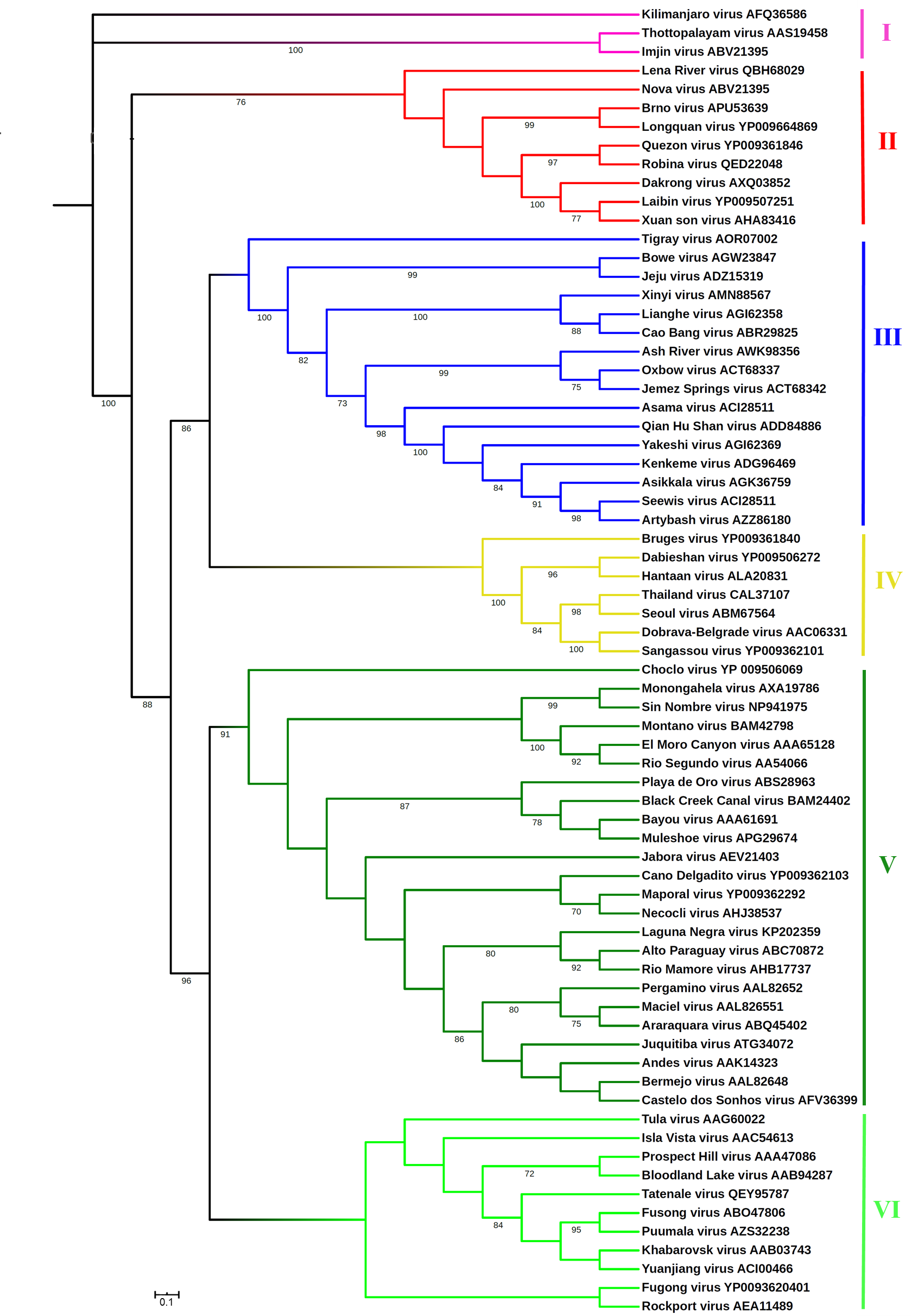

Supplement: Supplementary file 1 [file viruses-13-01188-s001.zip › Tree_Hanta_bats.tif]
